# Hindbrain and Spinal Cord Contributions to the Cutaneous Sensory Innervation of the Larval Zebrafish Pectoral Fin

**DOI:** 10.3389/fnana.2020.581821

**Published:** 2020-10-20

**Authors:** Katharine W. Henderson, Alexander Roche, Evdokia Menelaou, Melina E. Hale

**Affiliations:** Department of Organismal Biology and Anatomy, College of the University of Chicago, Chicago, IL, United States

**Keywords:** sensory systems, zebrafish, rohon-beard, pectoral fins, neuroanatomy

## Abstract

Vertebrate forelimbs contain arrays of sensory neuron fibers that transmit signals from the skin to the nervous system. We used the genetic toolkit and optical clarity of the larval zebrafish to conduct a live imaging study of the sensory neurons innervating the pectoral fin skin. Sensory neurons in both the hindbrain and the spinal cord innervate the fin, with most cells located in the hindbrain. The hindbrain somas are located in rhombomere seven/eight, laterally and dorsally displaced from the pectoral fin motor pool. The spinal cord somas are located in the most anterior part of the cord, aligned with myomere four. Single cell reconstructions were used to map afferent processes and compare the distributions of processes to soma locations. Reconstructions indicate that this sensory system breaks from the canonical somatotopic organization of sensory systems by lacking a clear organization with reference to fin region. Arborizations from a single cell branch widely over the skin, innervating the axial skin, lateral fin surface, and medial fin surface. The extensive branching over the fin and the surrounding axial surface suggests that these fin sensory neurons report on general conditions of the fin area rather than providing fine location specificity, as has been demonstrated in other vertebrate limbs. With neuron reconstructions that span the full primary afferent arborization from the soma to the peripheral cutaneous innervation, this neuroanatomical study describes a system of primary sensory neurons and lays the groundwork for future functional studies.

## Introduction

The limbs of vertebrates are innervated with primary sensory afferents that provide input on both movement of the limb and properties of the animal’s environment. In mammals, these sensory neurons are known to synapse directly with both local spinal motor neurons and ascending projection neurons relaying information to the brain (Azim et al., 2014; Zampieri et al., 2014; Hachisuka et al., 2016; Abraira et al., 2017). Like the skin of terrestrial mammalian limbs, the skin of aquatic vertebrate fins is also densely innervated by sensors (Hughes, 1957; Roberts and Hayes, 1977; Taylor and Roberts, 1983; Clarke et al., 1984). Fin sensory neurons transmit mechanosensory and other inputs from the fin (Lowenstein, 1956; Bardach and Case, 1965; Ridge, 1977; Silver and Finger, 1984; Williams Iv et al., 2013), and those inputs modulate movement (Williams and Hale, 2015; Aiello et al., 2020). To interpret fin mechanosensory physiology and function in behavior, a more detailed understanding of sensory neuroanatomy is required.

The larval zebrafish pectoral fin provides a complementary system to adult fish and tetrapods for examining vertebrate limb sensation. At 5 days post fertilization (dpf), the pectoral fins are flexible structures that are active during locomotor bouts and periodically when the fish is stationary (Thorsen et al., 2004; Green et al., 2011). At this late larval stage, the fin can be subdivided into two regions: the fin body and the fin membrane. The fin body (FB) includes an endochondral disk separating the fan-shaped adductor and abductor muscle on either side (Thorsen and Hale, 2005). Parallel to the endochondral disk, a simple set of muscles originate in the fin base and insert into the actinotrichia in the more peripheral fin membrane (FM), which eventually gives rise to the adult fin rays (Thorsen and Hale, 2005). A single blood vessel bounds the FB from the FM. Movement studies have shown that the fin has a distinct curvature at this blood vessel during fin abduction (Green et al., 2011, 2013). This articulation appears functionally analogous to the elbow joint of a tetrapod limb that allows bending in one direction.

The fins can beat rhythmically and have been shown to play a critical role in fluid mixing that occurs near the head and anterior trunk, thus supporting cutaneous respiration (Green et al., 2011, 2013). Movement of the fin with the pair of muscles at the FB is coordinated by a pool of pectoral fin motor neurons that innervate the FB (Thorsen et al., 2004; Thorsen and Hale, 2007). This motor population has mixed origins in both the spinal cord and the hindbrain, consistent with myomeres two through five (Myers, 1985; Ma et al., 2010). Adductor and abductor motor neurons present a mixed mediolateral distribution within the pectoral fin pool (Thorsen and Hale, 2007). These motor neurons give rise to the rhythmic asynchronous fin beats that encourage the mixing of the water immediately surrounding the fish. While prior work has indicated a sensory population innervating the fin (Thorsen and Hale, 2007), there has not been an in depth exploration of the corresponding sensory neurons.

For limb sensation in larval zebrafish and aquatic tetrapods, the focus of research has been on spinal cord sensory neurons. The primary neurons examined in larval zebrafish are called Rohon-Beard cells (RBs). Early research in *Xenopus laevis* characterized RBs as transient mechanosensory neurons that innervate the body axis and respond to touch (Rohon, 1885; Beard, 1889, 1892, 1896; Hughes, 1957; Roberts and Hayes, 1977; Taylor and Roberts, 1983; Clarke et al., 1984). These studies described RBs dying off during mid-larval stages (Hughes, 1957; Reyes et al., 2004). More recent work in zebrafish has found that RBs are not only intact at late larval to juvenile stages (Palanca et al., 2013; Williams and Ribera, 2020), they are also functioning as key touch responsive neurons in that species (Faucherre et al., 2013; Katz et al., 2020). At 5 dpf, RBs have established connections with motor circuits (Böhm et al., 2016; Hubbard et al., 2016; Umeda et al., 2016; Knafo et al., 2017; Liu and Hale, 2017). As a functional sensory population at 5 dpf, we hypothesized RBs would be innervating the pectoral fins. Like other vertebrates, larval zebrafish also possess dorsal root ganglion (DRG) neurons. Past hypotheses suggested that the timing of DRG neuron development also signaled RB apoptosis (Hughes, 1957), but this is now understood to be de-coupled from RB cell death (Reyes et al., 2004). Indeed, the two neuron types overlap during mid to late larval stages (Bernhardt et al., 1990; Williams et al., 2000; Honjo et al., 2008). By 48 hours post fertilization, the most anterior of the DRG neurons have begun to differentiate and migrate ventrally (Raible et al., 1992; Williams et al., 2000). Between two and half and three dpf, larval zebrafish have at least one fully differentiated DRG neuron present at the level of the ventral root of each myomere along the entire body axis (Bernhardt et al., 1990; Williams et al., 2000). The axons of DRG neurons and RBs together form the dorsal longitudinal fasciculus (DLF) (Bernhardt et al., 1990). While DRG neurons develop later in the post-embryonic stages, their anatomical distributions in each myomere and their projections mixing with RB axons suggest the possibility of similar targets within the spinal cord. DRG neurons steadily increase in number until there are over 100 neurons in a single ganglion at 28 dpf (An et al., 2002).

We present here the first in depth description of the afferent arborization of the whole larval zebrafish pectoral fin surface. Using the larval zebrafish model, we aimed to determine how sensory neurons innervating the skin of the pectoral fin are organized as a population in the central nervous system (CNS). The robust genetic toolkit available in the zebrafish (Asakawa and Kawakami, 2008) facilitates the neuroanatomy work presented here. In addition, 5 dpf zebrafish are small and transparent, thus allowing for *in vivo* approaches to study neuroanatomy. Here, we image the entire sensory innervation of the pectoral fin from CNS to peripheral skin. This *in vivo* preparation avoids distortion related to fixation and sample processing. Furthermore, we sought to assess afferent anatomy and how it relates to other aspects of fin anatomy, movement, and function.

In exploring the cutaneous innervation of the 5 dpf larval zebrafish fin, we sought to answer several central questions. First, what is the population identity of the fin sensory neurons (FSNs)? Based on prior work in the zebrafish motor system (Ma et al., 2010), we hypothesized that we would see a mixed population of hindbrain and spinal cord sensory neurons innervating the fin. Second, do the FSNs “map” to specific areas of the fin? Based on prior work in sea robin (Morrill, 1895; Finger, 1982) showing somatotopic organization of sensory innervation of the free fin rays, we anticipated that we would see some degree of somatotopy with FSN somas in the CNS organized according to their afferent patterns in the pectoral fins. We hypothesized that FSN somas would exhibit similar patterning. Based on prior data in axial RBs in zebrafish and *Xenopus laevis* (Roberts and Hayes, 1977), we also anticipated a high degree of branching of the primary afferents of each cell. Finally, we asked: is the innervation of the pectoral fin concentrated at the location of increased bending on the fin that occurs between the FB and the FM (Green et al., 2011, 2013)? Given the functional importance of this region, we expected some heterogeneity with greater innervation in the FM compared to the FB. In addition to describing sensory innervation, together with existing work on the motor system, these data support future studies exploring sensorimotor integration and fin function.

## Materials and Methods

### Fish

Animal use was approved by the University of Chicago’s Institutional Animal Care and Use Committee. Adult zebrafish were maintained at 27°C on a 14/10 h light/dark cycle in a custom fish facility. Fertilized eggs were held in 10% Hank’s Solution in a 28.5°C incubator until 5 dpf. At 5 dpf, larval zebrafish were used for imaging studies and then euthanized in 0.02% 3-aminobenzoic acid ethyl ester (MS222, Sigma-Aldrich, St. Louis, MO, United States). To target sensory neurons, we used two transgenic lines that drive reporter expression in *islet2B* + neurons (Pittman et al., 2008; Olesnicky et al., 2010). The *islet2B* lines label the sensory neurons in zebrafish, and these lines have been used in a number of studies targeting sensory neurons for comparison of expression patterns or for functional interrogation (Olesnicky et al., 2010; Knafo et al., 2017). For initial studies examining the whole population, we used the *Tg[isl2b:GFP]^*zc*7^* transgenic line (*Tg[islet2b:GFP])* (Pittman et al., 2008). In some cases, we used double transgenics by crossing *Tg[islet2b:GFP]* fish with *Tg[mnx1:Gal4;UAS:pTagRFP]* (Zelenchuk and Brusés, 2011; Bello-Rojas et al., 2019) fish to examine the motor pool innervating the fin (Ma et al., 2010). The *Tg[mnx1:Gal4;UAS:pTagRFP]* line exclusively labels motor neurons (Zelenchuk and Brusés, 2011). We subsequently used *Tg[islet2b:Gal4]*^*zc*60^ transgenic animals (gift from McLean Lab, Northwestern University, Evanston, IL, United States; Fredj et al., 2010) to achieve sparse stochastic labeling (Asakawa and Kawakami, 2008; Menelaou et al., 2014), enabling the reconstruction of individual neurons.

### Mauthner Cell Labeling

*Tg[islet2b:GFP]* fish at 4 dpf were briefly anesthetized in 0.02% MS222 in Hanks. Once fish were non-responsive to touch, we placed them on a petri dish filled with 2% agar. Using 10% dextran conjugated with Alexa Fluor 647 (10,000 MW Thermo Fisher Scientific, St. Louis, MO, United States), we followed the backfilling procedure described previously (Hale et al., 2001). In these experiments, the capillary tube was aligned to be parallel with the ventral boundary of the spinal cord of the 4 dpf larval zebrafish. Fish were allowed to recover post injections for 24 h so that the dextran could thoroughly permeate through backfilled neurons. Fish were then imaged on a Zeiss LSM 710 confocal microscope with a 20x dry 0.8NA objective.

### UAS Construct Injections

Embryos from *Tg[islet2b:Gal4]* transgenic fish were collected immediately following fertilization. These embryos were then transferred to an injection plate composed of a plastic dish with a microscopy slide taped to it. Using capillary action with a Kimwipe (Thermo Fisher Scientific, Waltham, MA, United States) on the opposite side, embryos were aligned against the slide. In this arrangement, embryos at the one or two-cell stage were held stationary for DNA construct injections to generate stochastic labeling in the *Tg[islet2b:Gal4]* line with UAS:ptagRFP as described previously (gift from McLean Lab, Northwestern University, Evanston, IL, United States; Zelenchuk and Brusés, 2011; Menelaou et al., 2014). Following injections, embryos were transferred to fresh 10% Hanks and maintained at 28.5°C in a Fisher Scientific Low Temperature Incubator (Thermo Fisher Scientific, Waltham, MA, United States). After hatching at 3 dpf, embryos were transferred to fresh Hanks and screened for single cell or sparse multi-cell labeling in the pectoral fin using a Leica MZFLIII dissecting scope (Leica Microsystems, Inc., Buffalo Grove, IL, United States) with a mercury lamp as the excitation source.

### Imaging

Fish were transferred to a 24-well plate to track left or right pectoral fin innervation. At 5 dpf, fish were anesthetized in 0.02% MS222 in Hanks, and mounted laterally in low-melt agarose as previously described (Hale et al., 2001). In this study, we used a round 35 mm dish with high precision No. 1.5 coverglass (MatTek Corporation, Ashland, MA, United States) for optimal signal. To facilitate imaging of processes in the area of the yolk sac, the air bubble from the swim bladder was carefully removed with a patch pipette secured to a 1 mL syringe with Parafilm. With this process, it was possible to remove the swim bladder from the non-imaging side of the fish with minimal disruption to the cells of interest. Embedded fish were imaged on a Leica TCS SP8 II STED laser scanning confocal microscope (Leica Microsystems, Inc., Buffalo Grove, IL, United States) under Köhler illumination conditions. We used the White Light Laser set to 555 nm as the excitation source, and the specimen was imaged through a 40x/1.30 NA oil immersion HC PlanApo objective. For RFP, the detector was a HyD tuned to 562–700 nm with gain set to 20 and gating turned on, and for visible light the detector was a PMT with gain set at 415. We used bidirectional scanning with phase adjusted at the beginning of each imaging session. Pinhole was set for 1 airy unit. For each fish, we imaged a 3 × 3 region with excitation gain turned on through a 150–250 micron z-stack for a total of nine stitched images of 1,024 × 1,024 pixels at 8-bit depth using LAS X software (Leica Microsystems, Inc., Buffalo Grove, IL, United States), stitched in the software with statistical blending between tiles, and saved as.lif files (see Supplementary Movie S1 for single whole cell volume).

### Soma Data

To calculate the size of single RB somas, we used the Bio-Formats (Linkert et al., 2010) importer in Fiji version 2.0.0-rc-71/1.52p, Java version 1.8.0_172 (Schindelin et al., 2012; Schneider et al., 2012) to open.lif z-stack files for image processing on a desktop computer with an Intel Zeon CPU E5-2630 v4 @ 2.20GHz x 10, 62.8GiB of memory, 2TB hard drive, and an NVIDIA Corporation GM107GL (Quadro K2200) graphics card (Dell Round Rock, TX, United States) running Linux Mint 18.3 Cinnamon 64-bit (Cinnamon version 3.6.7, Linux Kernel: 4.10.0-38-generic, everyone, everywhere). In the full z-stack, we selected the single micron optical section in the middle of the soma along the medial to lateral axis. Using the freehand selection tool in Fiji, we traced around the fluorescent boundary of the cell body and recorded the value.

### Innervation Reconstruction

Whole z-stack tile scans were processed in Fiji (Schindelin et al., 2012; Schneider et al., 2012) using the Tubeness plugin (Sato et al., 1998) with a sigma value of 1. Output images were saved as 32-bit depth.tif files. We note that these files require substantial memory to open and work with. Post preprocessing for “tubes,” we reconstructed the fin innervation of 21 FSNs using the semi-automated Simple Neurite Tracer (SNT, version 3.1.3; Longair et al., 2011). Due to overlapping processes, we were able to accurately reconstruct the axial portion of only 12 neurons of the original 21 FSNs. We reconstructed the fin innervation projections for 20 neurons. Fin afferent reconstruction was not performed on one RB since it did not branch in the fin. For detailed analyses of reconstructions, SNT.traces files were converted to .swc files and analyzed with a custom C script on a MacBook Air with macOS High Sierra 10.13.6 (Apple, Cuptertino, CA, United States). Traces were re-segmented to isolate the interbranch segments of each primary afferent and the following metrics were quantified for each neuron: (1) number of branch points, which is the total number of branches of the afferent in the fin and the processes leading up to the fin (if the processes branch before the fin), (2) maximum order of any branch on the tree, which is how many branch points (n) are between the soma and the end point of the most branched afferent (n + 1), (3) Strahler number, which is the degree of branching as calculated backward from each terminal branch as 1, subsequent more basal branches are n + 1 if both daughters are n, alternatively the next branch is also n if one daughter is n and the other is less than n, (4) average partition asymmetry over all of the branch points, which is the measure of asymmetry of the tree where 0 is completely symmetric and 1 is highly asymmetric, (5) maximum path distance from the soma to any terminal point, the longest afferent from the soma to the terminal point, (6) the average contraction of the neuron, the ratio of the euclidean distance of a path to the actual path length that generates one estimation of the space filling of a neuron, (7) the average angle of the branches near the branch point, which is the Euclidean distance of each segment divided by the total length of the neuron, (8) the average local angle, which is the immediate angle 10 nodes (pixels) away from the branch point in each segment, (9) the average remote angle, which is the angle 10 nodes (pixels) away on the next daughter branch, (10) the average fractal dimension of the branches, which is a measure of how much the afferent meanders with a straight line being one and a more meandering line having a slightly higher value than one, (11) total length of all segments, all of the lengths of the segments projecting to the fin added together from the initial branch off the soma to the entire innervation of the fin, (12) the area of the soma, which is calculated at a single micron z-slice from the confocal z-stack, and (13) the anteroposterior position of the soma, which is calculated with reference to the boundary between myomeres three and four (Ascoli et al., 2007; see Supplementary Tables S1, S2 for values and a full description of morphological parameters).

### Neuronal Classification

Based on the number of FSN RBs in the dataset, we opted to use a hierarchical clustering algorithm to examine the dissimilarities among the 20 reconstructed neurons that exhibited branching in the fin. Data were scaled in R (R version 3.6.3; R Core Team, 2014, RStudio Version 1.2.5042; RStudio, 2020) such that the mean over all neurons was 0 and the standard deviation was 1. A dissimilarity matrix based on the Euclidean distance of the individual measures of each of the 13 parameters was established. We used agglomerative hierarchical clustering using Ward’s linkage method (Ward, 1963) in R. In this method, individual neurons, the leaves, are iteratively combined into nodes based on the similarity between them. Grouping continues until all the leaves are part of one big cluster. We used the average silhouette method to confirm the optimal number of clusters. This method allows for intracluster evaluation of similarity, which is how well an individual fits within its cluster.

### Quantification of Axis and Fin Innervation

Even with sparse stochastic labeling, there was overlap of primary afferent arborizations on the axis that made it difficult to reconstruct individual neurons. Due to this overlap, we were restricted to full reconstructions of fin and axis innervation in only 12 neurons. All of them had their cell bodies in the hindbrain. When the fin is adducted it lies close to or against the axis. To examine the spatial relationship of the axial innervation relative to the fin innervation of a cell we examined how axial innervation and fin innervation overlap, and potentially interact, in this region. We designated a “model fin” that was used as a stereotyped fin across those sampled. A 2D projection of the model to its best-fit plane was rotated and moved for each fish such that the midpoint of its base and the angle of its base made with the body matched up to the actual fish’s fin. The axial innervation was projected onto the plane defined by this fin, and innervation within this area was assumed to be contacted by the fin during adduction. The total length of the innervation within this area, along with the percentage of the total axial innervation within this area, was calculated for each fish.

### Fin Quadrant Innervation

To analyze the innervation patterns within the fin, we divided the fin into eight regions. Lateral and medial surfaces were both examined. For each, the FM and FB were bisected into ventral and dorsal portions (see Figure 5A), generating a dorsal and ventral FM region and a dorsal and ventral FB region for both the lateral and medial surfaces. We used the Bio-Formats importer (Linkert et al., 2010) in Fiji (Schindelin et al., 2012; Schneider et al., 2012) to open the fluorescent and brightfield confocal z-stacks. We merged the two images together and used the multipoint function in Fiji to assign 18 points in three-dimensional space: nine points on the outer edge of the FB as demarked by the blood vessel and nine points on the edge of the FM. Of each of these sets of nine points, two points were at the base, one on either side, one point was at the maximum distance from the base, and, on each side of that point, there were three points that roughly defined the shape of the fin. In Mathematica version 12.0.0.0 (Wolfram Research, Champaign, IL, United States), we used the 14 non-base points to find a best-fit plane onto which we orthogonally projected all of the points. We also projected each of the 21 reconstructed neurons onto this plane, one at a time. We divided the plane into quadrants based on the fin points: the dorsal and ventral portions of FB and FM. A line from the midpoint of the proximal and distal points of the fin body to the median point on the fin membrane defined the dorsal/ventral boundary (Figure 5A). In SNT, we tagged the primary afferents to segregate them into medial surface innervation and lateral surface innervation. Altogether, we had eight different possible fin locations. In Mathematica, we calculated the total length of each primary afferent in each of these octants.

### Figure Preparation

We generated a duplicate set of microscopy images specifically for image preparation. Stitched, tiled z-stacks were projected along the z-axis, smoothed in Fiji, and flattened. We generated merged two channel images as well as z depth color coded single channel images. When necessary, we cropped processed images for specific regions of interest. All graphs were generated in RStudio using ggplot2 (Wickham, 2016). All figures were arranged in Adobe Illustrator (Adobe, San Jose, CA, United States).

## Results

### Pectoral Fin Surfaces Are Innervated by Projections of Neurons From the Hindbrain and Spinal Cord

**FIGURE 1:**
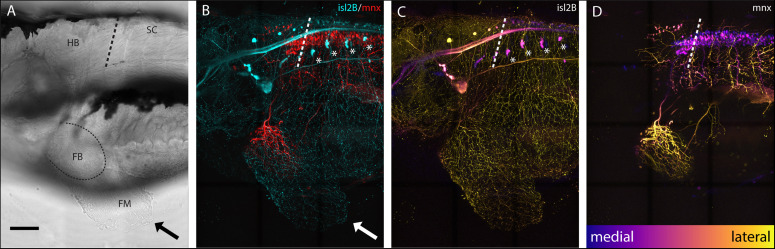
islet2B + neurons innervate the pectoral fins of 5 dpf larval zebrafish. **(A)** Brightfield lateral view of a 5 dpf larval zebrafish. Arrow indicates distal fin membrane, and dotted line indicates the blood vessel boundary between the fin body (FB) and fin membrane (FM). **(B)** *Tg[islet2B:GFP]* × *Tg[mnx1:Gal4;UAS:pTagRFP]* double transgenic fish (*N* = 4) showing sensory neurons and their processes (cyan) and motor neurons and their processes (red). Arrow indicates distal fin membrane. Asterisks indicate DRGs. **(C)** Depth coded z-projection of *islet2B* (neurons highlights the lateral placement of the sensory neuron cell bodies compared to the more medially located mnx1+ motor neurons in **(D)**. Asterisks indicated DRGs. Depth scale is the same for **(C,D)**. Anterior is to the left, dorsal is up in all images. Scale is 100 microns.

**FIGURE 2:**
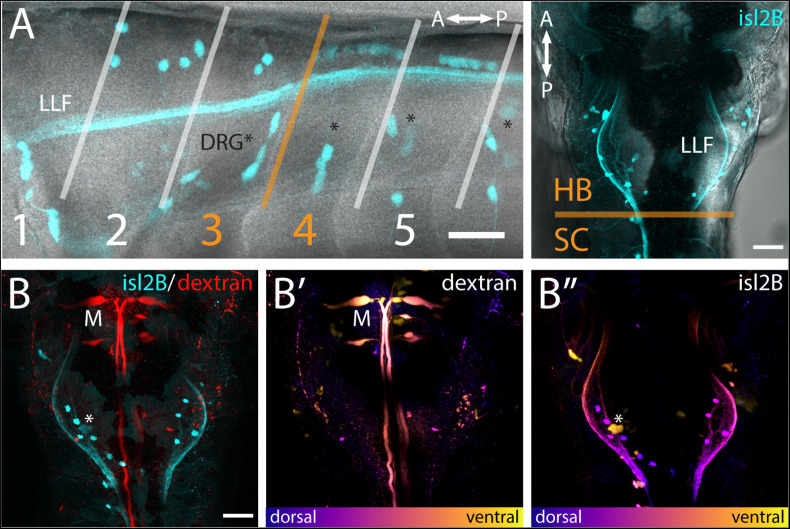
FSNs have somas in the hindbrain and spinal cord. **(A)** Left panel: Brightfield and fluorescent merged image adjacent to myomeres one through five, indicating the distribution of the *islet2B* + somas (cyan) innervating the fin. The FSN somas innervating the fin are located dorsal to the dorsal root ganglion (DRG) neurons visible as clusters (asterisks). Fibers from DRGs also projected to the fin at this stage, but they cannot be traced individually. The lateral longitudinal fasciculus (LLF) extends from the hindbrain into the spinal cord. The transition area between myomeres three and four, historically referred to as the transition between the hindbrain and spinal cord, is indicated in orange. Cells posterior to the boundary are located in the spinal cord, and *islet2B* + cells in this location are Rohon-Beards. Right panel: Brightfield and fluorescent merged image of a dorsal view of a *Tg[islet2B:GFP]* fish. The hindbrain and spinal cord boundary is again marked in orange. The organization of the sensory neurons along the lateral margins of rhombomere 7/8 is bounded by the LLF. **(B)** All of the somas (cyan, left panel) are located posterior to the Mauthner neurons (red, M). Additionally, Mauthner neurons are notably more ventral (**B′**, M) in the hindbrain than the FSNs (**B″**, asterisk is DRG). Anterior is to the left, dorsal is up in (**A** left panel). Anterior is up, dorsal view in (**A** right panel and **B**). Scale is 40 microns in (**A** left panel) and 50 microns in (**A** right panel and **B**).

Sensory neurons, both DRGs and FSNs, labeled in *Tg[islet2b:GFP]* transgenic fish have somas in the hindbrain and spinal cord area, and their processes can be seen innervating the pectoral fin (Figure 1A brightfield compared to Figure 1B). This transgenic line, labeling the sensory neurons, was used to examine the broader features and locations of the somas of fin-innervating cells. We used the *Tg[islet2b:GFP]* x *Tg[mnx1:Gal4;UAS:pTagRFP]* double transgenic fish to examine the sensory neuron cell bodies relative to motor pools in the CNS (Figure 1C vs. Figure 1D). There are two distinct populations of motor neurons visible in the z-projected z-stack: a spinal population and a hindbrain population (Figure 1D) shown previously (Ma et al., 2010). The number of spinal cord motor neurons appears larger than the number of hindbrain motor neurons (Figure 1D). We find that the FSNs are distributed similarly with both a hindbrain and spinal cord population. The hindbrain FSNs are located more lateral and dorsal with respect to the motor neuron population (Figure 1C compared to Figure 1D). In the spinal cord, dorsally located FSNs are consistent with Rohon-Beard neurons (RBs) (Figure 2A, *n* = 4), and their processes innervating the fins extended from the RB pool associated with myomere four and five. The total number of RB somas associated with myomeres four and five ranged from six to eight (*n* = 4).

The hindbrain component of the FSN pool was anterior to the boundary between muscle myomeres three and four (orange line, Figure 2A) and located in hindbrain rhombomere 7/8. In the hindbrain FSN population, a minimum of two cell bodies (not shown) and a maximum of seven cell bodies were labeled per fish (Figure 2A) (n = 14 fish). To refine information regarding the location of the hindbrain fin sensory neurons, they were examined in *Tg[islet2b:GFP]* fish that had been secondarily labeled through injection of dextran to fill reticulospinal cells (Figure 2B; *n* = 10). The most anterior *islet2B* + neuron in each fish was on average 184.38 ± 8.83 μm (average ± SE) posterior to the Mauthner neurons, which are located in rhombomere 4 (Kimmel et al., 1982). Mauthner neurons are on average 279.82 ± 7.93 μm from the HB/SC boundary, as previously defined (Morin-Kensicki et al., 2002; Ma et al., 2009, 2010), confirming that these fin neurons are well within the hindbrain. In lateral view (Figure 2A, left), FSNs show a range of dorsoventral positions. In dorsal view, labeling of the lateral longitudinal fasciculus (LLF), provides a marker along the mediolateral axis (Figure 2A, right). The fin sensory neurons that we observed were all located medial and dorsal to the LLF and dorsal to the level of Mauthner neurons (Figures 2B′,B″).

### Fin Sensory Neurons Show a Variety of Soma Morphologies

**FIGURE 3:**
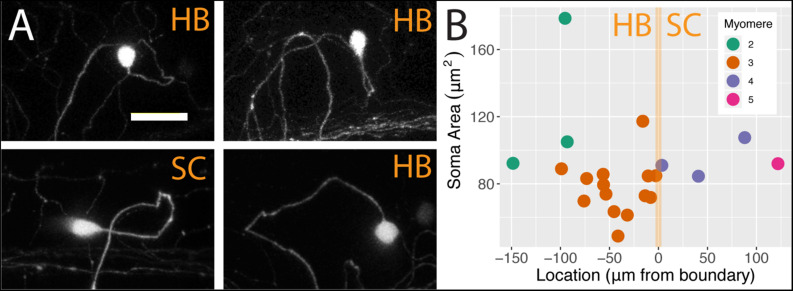
FSNs exhibit a variety of soma morphologies. **(A)** The four main FSN soma morphologies clockwise from top left: small spherical hindbrain (HB), small teardrop HB, rounded HB with dorsal projection, and a classic dorsally located RB with its dorsoventrally compressed shape resulting in elongation along the anteroposterior axis. The three hindbrain morphologies are apparent across cells associated with myomeres two and three. **(B)** FSNs are distributed across myomeres two through five, and they show no significant trends in soma size or distribution patterns across the anterior to posterior axis within any of these myomeres. FSNs exhibit no trends with regard to the hindbrain/spinal cord transition area (indicated in orange). Negative numbers are consistent with a hindbrain location, and positive numbers indicate a spinal cord location. Most of the cells innervating the fin are found in myomere three. Anterior is to the left, dorsal is up in **(A)**. Scale is 25 microns in **(A)**.

We analyzed individual neurons in 21 sparsely labeled *Tg[islet2b:Gal4]* larval fish injected with UAS:ptagRFP, again using confocal microscopy. In these stochastic labeling experiments, a large majority of the cells (17 out of 21) were located in the hindbrain. The remaining cells were RB neurons in the spinal cord, of which three were associated with myomere four and only one was associated with myomere 5. The population of FSNs sampled exhibited a range of morphologies. At the level of myomere two, a total of three neurons were labeled. One of the cells was circular and 178.60 μm^2^, another was asymmetric, spherical, and 105.03 μm^2^, and the third was teardrop shaped and smaller at 92.24 μm^2^ (Figure 3A, clockwise second image). In myomere three, where 14 cells were labeled, we observed seven spherical cells ranging from 61.39 to 117.31 μm^2^, four teardrop shaped cells ranging from 48.9 to 88.95 μm^2^, and three inverted teardrop shaped cells that were 73.82, 79.44, and 85.64 μm^2^ (Figure 3A, clockwise third image). In contrast, all of the spinal neurons at the levels of myomeres four and five (*n* = 4) exhibited the classic morphology of RBs: they were elongated along the anteroposterior axis, dorsally displaced, and arranged in a columnar fashion in the dorsal most regions of the spinal cord (Figure 3A, clockwise fourth image). RBs ranged from 84.56 to 107.56 μm^2^ in cell area (mean 93.77 = μm^2^, *SD* = 9.76 μm^2^, Figure 3B). There is no significant difference in the areas of HB FSNs and RB FSNs (Figure 3B, ANOVA, *p* > 0.05).

### FSNs Innervate the Axis and Extend Concomitant Projections to the Fin

**FIGURE 4:**
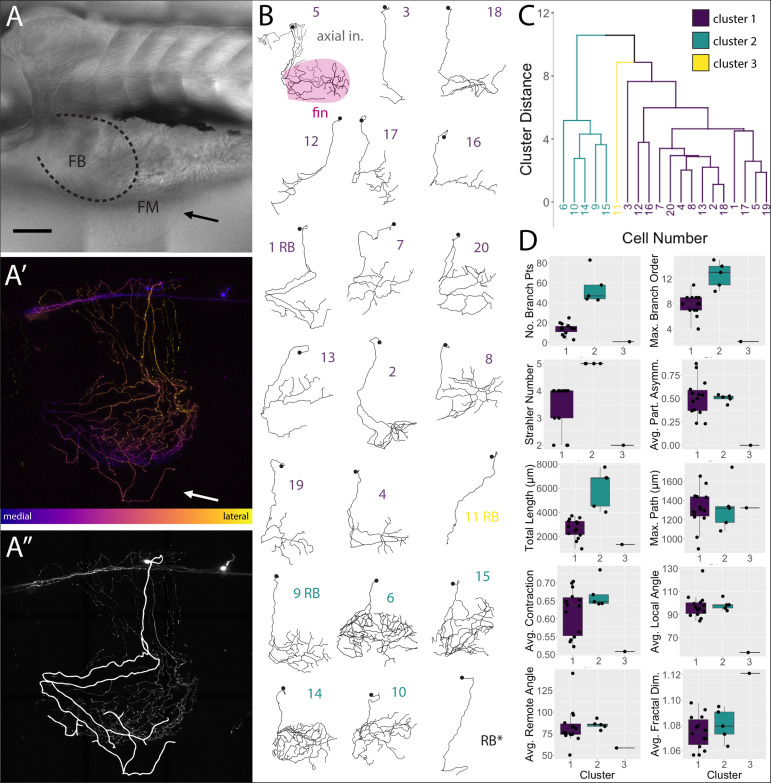
*islet2B* + neurons innervating the fin fall into three distinct morphological clusters. **(A)** Brightfield image of 5 dpf larval zebrafish in lateral view. The fin is indicated with a black arrow, and the fin body (FB) is bounded by a dotted line indicating the position of the blood vessel separating the FB and the fin membrane (FM). **(A′)** A depth coded z-projection of a single *islet2B+*neuron from the same fish in **(A)** shows an extensive arborization on the body and some sparse processes visible in the fin (white arrow). **(A″)** The same z-projection with an overlay of the reconstruction of the primary afferents projecting into the fin. **(B)** Reconstructions of the primary afferents innervating the fin show a diversity in both innervation pattern and fin coverage. The reconstruction in the top left has the axial innervation included in gray, the rest of the reconstructions are of only the fin innervation. The numbers are color coded to correspond to one of the three clusters in **(C)**. **(C)** Dendrogram of the results of agglomerative hierarchical clustering analysis using Ward’s method. There are three distinct clusters, color coded to reflect the number labels associated with the neuronal reconstructions in **(B)**. The y-axis indicates the Euclidean distance between clusters and leaves. **(D)** Box plots of each of the 10 morphological parameters, together with two soma parameters from Figure 3, utilized in the cluster analysis. Boxes are color coded in accordance with cluster number, with the exception of cluster 3, which only contains one cell. Black point overlays indicate the individual values for each neuron. Anterior is to the left, dorsal is up in **(A,A′,A″).** Scale is 100 microns in **(A)**. *N* = 21 in **(B)** and 20 in **(C,D)**.

FSNs projected toward the fin in two different manners. At this stage in development (5 dpf), the pectoral fins are comprised of fan-shaped musculature in the fin body (FB) and a surrounding thin cutaneous fin membrane (FM) (Figure 4A). The processes of the FSNs exit the CNS and establish extensive innervation projections along the axis and into the fin (Figure 4A′). All 21 of these neurons showed extensive innervation across the skin of the axis. At the level of the skin, FSN processes track to the fin body and enter the fin in two possible configurations. In five out of 21 cases, FSNs had two branches that eventually projected into the fin (Figure 4A″) whereas the majority of the reconstructed neurons (16 out of 21) had a prominent process that projected to the fin with no secondary projections that concomitantly entered the fin (Figure 4B).

Once the FSNs innervated the fin, they tended to project to fill the entire fin on both the medial and lateral sides. In preliminary examination, initial projections entering the pectoral fin were present on both the medial and lateral sides of the FB. Specifically, 19 of the 21 sampled neurons had initial projections onto the medial surface of fin while 16 of the neurons had initial projections onto the lateral surface of the fin. In the majority of neurons examined, the individual neurons innervated both sides of the fin. Afferents of many neurons wrapped around the fin membrane to innervate the opposite side. In two cases, reconstructed neurons innervated the fin, looped around and exited the fin. While the processes crossed over the surface area of the muscle in the FB, we never observed stochastically labeled cells with fiber endings in the FB muscle. The primary afferents exhibited occasional varicosities along their superficial primary afferents, but they lacked any obvious associated sensory structures, ultimately terminating in free nerve endings.

### FSNs Form Three Clusters Based on Morphological Parameters

We deconstructed the primary afferent reconstructions into individual branch components and analyzed 11 tree morphological parameters and two soma parameters (Supplementary Tables S1, S2 for elaboration on parameters). With these 13 parameters, we explored potential subtype classifications among the 20 individually reconstructed neurons with hierarchical clustering analysis (Figures 4C,D). As stated previously, the 21st neuron was eliminated due to its lack of any branches in the fin. Three clusters of neurons emerged: one group of highly arborized fin specific processes, a second group with less dense branching, and a third group containing a single neuron that has very limited branching in the fin. The first group contains 14 neurons that are spread across four arbitrary units (purple, Figures 4B,C). This group has a variety of neuronal morphologies represented (Figure 4C), with some cells exhibiting distally biased process branching (cell 2 in purple cluster one) and others exhibiting more even branching (cell 20 in purple). The second group contains five closely related cells (green, Figures 4B,C). In contrast to the first cluster, this second cluster (cells 6, 10, 14, 9, and 15 in green cluster two) exhibits higher degrees of branching and larger innervation lengths (Figure 4D). The third group, with just a single cell, is noticeably disparate from either cluster (Figure 4B, cell 11 in yellow cluster three). Differences in the number of FSNs in each cluster could reflect distributions of subtypes. Notably, HB and RB FSNs are intermixed in clusters one and two. The cluster arrangements remained when individual morphological components were removed and the clustering analysis was re-examined (e.g., removal of Strahler number values had minor effects on the cluster organization).

### Primary Afferents Are Not “Mapped” to FB or FM, but do “Map” to the Mediolateral Surfaces

**FIGURE 5:**
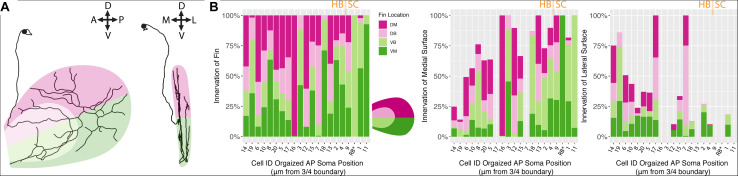
Fin sensory neurons exhibit biases to specific fin areas depending on soma location. **(A)** A single neuron reconstruction shows coverage of all four quadrants (left panel), and also shows bias toward the medial surface of the fin (right panel). Note: due to the nature of the projection in the right panel, the overlay of the dorsal and ventral shadings is consistent with the shading at the level of the blood vessel. **(B)** The distribution of primary processes across the fin quadrants including the dorsal membrane (DM, dark pink), dorsal base (DB, light pink), ventral base (VB, light green), and ventral membrane (VM, dark green) (left panel). Notably, the highest percentage of the total length of fibers is found in the medial fin surface (middle panel) across all quadrants compared to the lateral fin (right panel). Soma position values corresponding to the Cell IDs are detailed in Supplementary Table S3.

We hypothesized that, between the two main clusters of neurons, there may be distinct innervation patterns with reference to the pectoral fins themselves. Specifically, we sought to answer whether or not the FSNs exhibited any evidence of a fin “map,” with specific projections to certain areas. FSNs of the two clusters innervated the pectoral fins in a seemingly random manner (ANOVA, *p* > 0.05). Given the organization of the FSN population across the hindbrain and the spinal cord, we next examined the anteroposterior organization of the peripheral processes with regards to the total length of fin innervation. FSN clusters were not reflected across the anteroposterior location of somas, however we found a general trend across all three clusters as group. More anteriorly located FSNs had higher total afferent lengths in the fin than their more posterior counterparts (ANOVA, *p* = 0.04). Due to the longer initial axial segment leading to the fin projecting to the RBs, we re-examined these data without the RBs. This trend remains significant without the RBs (ANOVA, *p* = 0.031). Based on this trend in overall afferent length, we sought to examine any potential “map” arrangements in the fin. Here, we describe innervation patterns established along the dorsal and ventral part of the fin, pink for dorsal and green for ventral (Figure 5A). We also delineate between the FB and the FM, light pink and dark pink, respectively. By rotating the reconstructions, we could further subdivide the quadrants into octants based on their positioning on the medial or lateral surface of the fin (Figure 5A, right panel).

In the four main quadrants (the ventral and dorsal FB and the ventral and dorsal FM), there was no clear preference for one section over the other (Figure 5B). Primary afferents did not preferentially innervate the FM, as hypothesized, and they did not preferentially innervate the FB in either the dorsal or ventral regions (ANOVA, *p* > 0.05, Figure 5B). There were two cells, cell ID 16 and cell ID 1 RB, that had highly biased innervation patterns, one innervating almost exclusively the dorsal membrane and one innervating almost exclusively the ventral membrane and body (Figure 5B). In all the other cases, the distribution of the primary afferents across the fin quadrants lacked any clear organizational pattern. In no cases did we observe an obvious preference for the “joint” region that bends between the FB and the FM. These trends were consistent across the three clusters of FSNs. As stated above, the logical next investigation was to examine the functional octants of the pectoral fin.

Despite the lack of notable organization with respect to the FB or the FM, or the “joint” formed between the two areas, there was a trend in innervation of the mediolateral surfaces of the fin. Most of the primary afferent innervation in the fin was on the medial surface of the fin (Figure 5B middle panel), and we found a trend for higher total length of innervation on the medial surface of the fin. Correspondingly, the more posteriorly located FSNs innervated the lateral surface less (ANOVA, *p* < 0.01, Figure 5B, middle panel). This relationship was maintained even when we removed RBs from the dataset. The mediolateral innervation bias reflects a broader trend that we examine further below.

### Sensory Neurons Innervate the Body Wall as Well as the Fin

**FIGURE 6:**
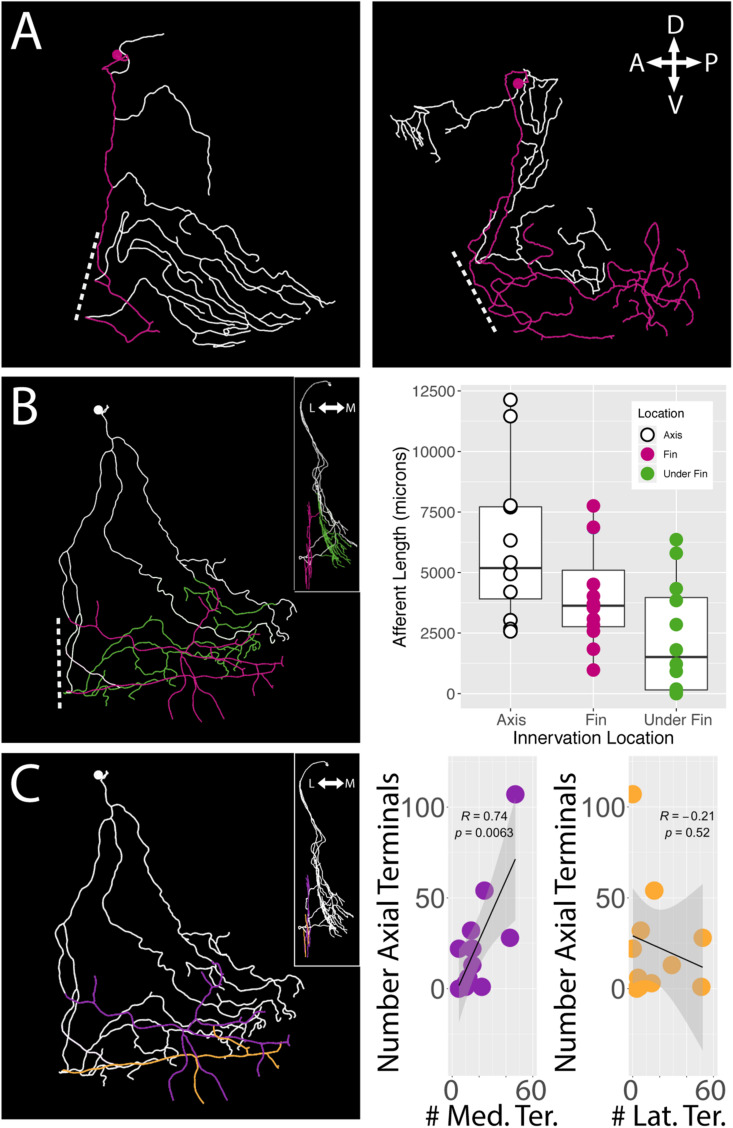
*islet2B*+ neurons innervate both the fin and the body axis. A subset of single neuron reconstructions color coded to indicate the axial innervation in white and the fin only innervation in pink. **(A)** *Left*, the cell has very little fin innervation and the primary afferent that enters the fin loops back out of the fin. *Right*, the cell has far more extensive coverage of the fin. **(B)** *Left*, a third cell is color coded for axial innervation (white), fin innervation (pink), and axial innervation under the fin (green). The inset image shows a 90 degree rotation of the same fish with the same color coding. *Right*, there is a substantial amount of axial innervation across all sampled fish, and the amount of axial, fin, or under the fin innervation varies across a wide range of afferent lengths. In general, the whole population has higher primary afferent lengths on the axis compared to the fin. Notably, some fish have zero to very little innervation under the fin. **(C)** *Left*, the same reconstruction in **(B)** is color coded for medial fin innervation (purple) and lateral fin innervation (orange). The inset again shows the same reconstruction rotated 90 degrees. *Right*, the number of terminals on the medial fin surface is positively correlated with the number of terminals on the axial surface under the fin (purple graph, correlation, *p* < 0.01). The number of terminals on the lateral fin surface shows no trend in relation to the number of terminals on the axial surface under the fin (orange graph, correlation, *p* > 0.05). Anterior is to the left, dorsal is up in reconstructions. In insets, medial is to the left and dorsal is up.

Regardless of their soma locations, all stochastically labeled cells examined innervated the skin of the body wall as well as the fin. In the interest of exploring the relationship between the fin surface and the axial surface, we reconstructed axial branches on 12 of the 21 FSNs that had no intermingled primary afferents with other stochastically labeled cells on the axial skin (Supplementary Figure S1). The lack of overlap allowed unambiguous assignment of the processes to the FSN of interest. These 12 neurons exhibited innervation both posterior to and anterior to the base of the fin (Figure 6A). In many cases, we observed innervation on the body axis under the fin (Figure 6B). The extent of axial innervation was comparable to that of the fin, with no significant difference between the total afferent length innervating the axial skin compared to the total afferent length innervating the pectoral fin surface (Figure 6B, right panel, Chi-square test, *p* > 0.05).

We examined the number of terminals on the medial and lateral surface of the fin as well as on the axis under the fin. As with the innervation percentages in the octants, we found a significant trend in mediolateral organization of terminals. We found that the number of medial surface fin terminals correlates positively with the number of terminals on the axial surface under the fin (Pearson’s correlation, *p* < 0.01) (Figure 6C, right panel left graph in purple). In contrast, no significant relationship was apparent between lateral surface fin terminal numbers and the number of terminals on the axial surface under the fin (*p* > 0.05) (Figure 6C, right panel right graph in orange).

## Discussion

The small size and transparency of the larval zebrafish and the molecular tools available to interrogate neuronal morphology make it possible to take a whole-structure approach to understanding limb innervation in a vertebrate. Here, we describe the sensory innervation of the pectoral fin, a vertebrate forelimb homolog. We show that the 5 dpf larval zebrafish pectoral fin is innervated by both hindbrain and spinal cord FSNs. The morphology of the spinal cord neurons is consistent with that of RBs. The HB FSNs appear distinct from the classic RBs based on both their anteroposterior position and cell body morphology. Our finding of both hindbrain and spinal sensory innervation builds on prior work describing the somas of the pectoral fin motor pool in both the hindbrain and the spinal cord (Ma et al., 2010). Prior work in the sea robin, *Prionotus carolinus*, showed that their unique fin chemosensory system had nerves originating solely in the spinal cord from specialized accessory spinal lobes that are formed by fusion of the DRG with the dorsal horn (Finger, 1982; Silver and Finger, 1984). Importantly, the work in *Prionotus* is indicative of the organization of sensory nerves during adulthood. Our results highlight a mixed sensory neuron population, demonstrating more complexity in the organization of sensory fin neuron populations across fish species and/or life stages than previously recognized. Additionally, the transience of RBs suggests that at least one component of the FSNs may change through ontogeny. The highly varied arborization patterns and the extensive innervation of both the fin and body wall has similar organization and appearance to work examining the peripheral sensory innervation of the enteric system of mammals (Spencer et al., 2014, 2018). Altogether, our findings suggest a lack of specificity for regions of the fin, thus raising questions regarding the function of the zebrafish FSNs. Future functional studies may be able to tease apart the relationship of our neuroanatomical findings to cellular function.

### Functional Implications of Morphological Features

The overwhelming majority of fin neurons labeled were located in rhombomere 7/8 (r7/8) of the hindbrain. We define r7/8 by its alignment with muscle myomeres one through three, and it is the most posterior of hindbrain rhombomeres. Accordingly, this region of the brain is in close proximity to spinal neurons (Prince et al., 1998; Skromne et al., 2007; Ma et al., 2009; Chang et al., 2016), but it is considered anatomically and functionally distinct. The portion of the axis immediately posterior to r7/8 is consistent with myomeres three and four (Ma et al., 2010), and in prior work this transition area between the HB and spinal cord has been referred to as a “boundary.” In adults of some fish species, DRGs in this region are fused to the spinal cord (Finger, 1982). As zebrafish are Cypriniforms, an order of teleost fish possessing a unique vocal apparatus by adulthood, it is important to note that this hindbrain region is the location of inferior olive neurons, respiratory neurons, and vocal pacemaker neurons (Bass et al., 2008; Ma et al., 2009; Bass and Chagnaud, 2012). Throughout development, the region between myomeres one through six undergoes a number of developmental changes as the vocalization function appears behaviorally (Morin-Kensicki et al., 2002). These features suggest a number of potential targets for hindbrain FSNs.

The variability in cell number observed in the hindbrain FSNs labeled in the (*Tg[islet2b:GFP])* is interesting with respect to canonical descriptions of other hindbrain neurons, like the reticulospinal neuron array, which is much more stereotypical in morphology and location of individual neurons (Kimmel et al., 1982). It is possible that there is some variability innate to this particular transgenic line. As previously described in sensory neuron populations, different enhancers drive expression at different times and in the same population of cells (Palanca et al., 2013). Alternatively, there are some other potential factors that could be leading to the variable numbers. First, the 5 dpf larval zebrafish is at the beginning of a transition stage in ontogeny during which the free-swimming larvae are beginning to hunt and feed. The larval fish pectoral fins are undergoing extensive growth and development (Thorsen and Hale, 2005), and, as a result, there is likely significant remodeling of the sensory system during this period. Second, at around 7 dpf, larval zebrafish begin to transition from cutaneous respiration to gill based respiration. It is possible that, as the 5 dpf larva progresses through the life history stages that require different sensory feedback and new motor repertoires, the sensory system must change to accommodate these needs. Additionally, perhaps variability in zebrafish neuron population numbers is not uncommon, as adult zebrafish exhibit widespread adult neurogenesis (Zupanc et al., 2005; Hinsch and Zupanc, 2007). On top of this, RBs have been described as a transient sensory neuron population that is lost during the larval stages as dorsal root ganglion neurons (DRGs) overtake some of their functions (Won et al., 2012). There is evidence that numbers of neurons are variable in a variety of organisms (Kollros and McMurray, 1955; Macagno, 1980; Kollros and Thiesse, 1985; Williams et al., 1996; Strom and Williams, 1998; reviewed in Williams and Herrup, 1988; Keller et al., 2018). In addition, adult neurogenesis in zebrafish results in about 6000 new neurons in the brain every 20 min (Hinsch and Zupanc, 2007). If the FSNs are in a similar period of transition, then it logically follows that we would observe variable numbers of FSNs reflecting the changing life history demands. Perhaps during this phase of rapid and extensive remodeling, the FSN population is undergoing equivalent changes. Furthermore, DRG neurons innervate the pectoral fin beginning at this stage (data not shown). This developmental timing, while not functionally linked to the turnover of RBs (Williams et al., 2000), may indicate a transitional period in the sensory architecture of the pectoral fin.

We found that fin sensory neurons of larval zebrafish also innervated the body wall adjacent to the fin. The number of terminal branches on the medial fin surface is positively correlated with those of the axial skin under the fin. In this context, our results suggest that, while somatotopic organization of fin sensory systems is apparent only at later stages in DRGs (Finger, 1982), trends in mediolateral terminal distributions may already be in place at larval stages. Future studies could further explore these trends across ontogeny, and functional studies could unravel the physiological importance of the mediolateral bias. Or, more simply, perhaps this organization is an early transition stage building the framework for a later anteroposterior organization. Prior work has examined the anteroposterior organization of fin motor nerves innervating both the larval and the adult zebrafish fin (Thorsen and Hale, 2007). In other specialized fish species, such as the sea robin (Finger, 1982) there is a similar organization of specialized accessory spinal lobes that are formed by fusion of the DRG with the dorsal horn. Importantly, these later stage fin nerve descriptions are most likely from DRGs, as is described in the sea robin work. Regeneration studies have identified non-neuronal cell types as critical landmarks for primary afferent regeneration after injury (Villegas et al., 2012). Perhaps trends in mediolateral terminal distributions are early drivers or landmarks for later developing sensory neurons that exhibit somatotopic organization along the anteroposterior axis.

That the FSNs of larval zebrafish innervate the skin of both the pectoral fins and the body axis suggests that they are not well suited for proprioception in the fin, as has been proposed for fin sensation in mature fish (Williams Iv et al., 2013). If the free nerve endings of the FSNs are mechanosensitive, they would be activating their own processes both in the fin and under the fin. Instead, it appears that these FSNs are likely to be non-specifically detecting stimuli across a large area of the larval body and pectoral fin. In this context, it seems unlikely that the FSNs would be activated by somatosensory stimuli specific for the fin itself. If they were, these cells would presumably be activated constantly as the fins are actuated. Instead, we propose that these limb sensors may be involved in chemosensory function related to cutaneous respiration, or they may be functioning as general rhythm detectors. The distribution of terminals across the fin, and in particular the correlation between medial fin surface terminal numbers and the under the fin surface terminals suggests a more generalized function of these sensory neurons. Prior work has established r8 as the location of rhythmic motor neurons (Bass et al., 2008; Ma et al., 2009), and the role of rhythmic pectoral fin movements in fluid mixing has been established (Green et al., 2011, 2013). A sensory population in this region would provide feedback on the nature of rhythmic systems critical for cutaneous respiration. Future functional studies could investigate these hypotheses in order to untangle the functional role of the FSNs.

Functional investigation would be particularly insightful within the fin. At later stages of ontogeny, the FM will eventually give rise to the fin rays (Grandel and Schulte-Merker, 1998), which we know from prior work encode proprioceptive feedback on fin ray bending in a number of species (Williams Iv et al., 2013; Aiello et al., 2016, 2017; Hardy et al., 2016). In sea robins, fin rays have also been shown to provide chemosensory feedback (Finger, 1982; Silver and Finger, 1984) and this is also likely to be more common than previously appreciated (Hardy and Hale, 2020). Subtypes in trigeminal neurons have been described at the morphological level (Pan et al., 2012), and *trpa1b* has been identified as an important channel in RBs regulating the response to nociceptive chemical stimuli (Prober et al., 2008; Gau et al., 2013). Taken together, we propose that the FSNs of larval zebrafish are involved in different sensory processes beyond mechanosensation, the known feature of RBs.

### Sensorimotor Remodeling Across Ontogeny and the Fin to Limb Transition

Prior work has shown that RBs are a transient population that begins to die off at some point during late larval stages. Initial reports suggested RB cell death began around 5–7 dpf (Williams et al., 2000). However, more recent studies have found that at least a subset of RBs are present at later stages, at least until 2 weeks post fertlization (Palanca et al., 2013; Williams and Ribera, 2020). In our lab, RBs have been anecdotally observed until at least 14 dpf (personal communication). Thus, the FSNs in both the hindbrain and spinal cord are possibly intact throughout the larval ontogenic changes to the fin. It will be necessary in future studies to explicitly identify the fate of these cells through ontogeny. If they are a transient population like RBs, there will be a need to determine when they die off and what sensory population replaces them.

The organization of RB and HB FSNs will need to be thoroughly investigated both across ontogeny and across fish species. In sturgeon, RB somas have been described in the caudal portion of the hindbrain (Kuratani et al., 2000). That work, together with our work presented here on the mixed population of HB and RB FSNs with similar soma sizes, suggests that there may be intermingling between these two cell populations. Alternatively, HB FSNs may represent an anteriorly displaced population of RBs. Teasing apart the differences between the two populations will require additional research. Honing in on the functional and morphological changes during ontogeny could shed light on the structural and functional changes of sensory systems across evolution. Teleost fish have developed a number of ways to repurpose paired fins for specific environmental needs (Finger, 1982; Silver and Finger, 1984; Murata et al., 2010; Aiello et al., 2017; Larouche et al., 2017), and there is plentiful evidence for variance in brain organization and structure across evolution (Ronan and Northcutt, 1990; Muñoz et al., 1997). Prior work in evolutionary developmental biology has indicated that the shift of pectoral fin motor neurons from a mixed hindbrain and spinal cord population to only a spinal cord population, as found in mammals, has largely happened as a result of a shift in Hox gene expression (Prince et al., 1998; Skromne et al., 2007; Ma et al., 2009; Chang et al., 2016). We have found that the sensory system in larval zebrafish follows the same pattern as the motor system, and the genetic patterning responsible for this will be interesting to investigate. A full description of the sensorimotor patterning in the zebrafish, together with the plentiful genetic tools available, could provide a playground within which to explore how the sensorimotor system of paired appendages can be remodeled.

Interestingly, this is the first time that sensory neurons from the hindbrain have been described innervating paired forelimb appendages. The sensory neuron organization we have described, from both the hindbrain and spinal cord, could represent an ancestral state, or it could represent a highly derived state that appeared at some point on the teleost lineage. The ability of the sensory neurons to exist between both the hindbrain and spinal cord organization suggests some degree of modularity, at least at larval stages. Prior comparative research across cyclostome species has indicated a high degree of variability within sensory structures (Ronan and Northcutt, 1990). Regardless of its origins in evolutionary history, we propose that further work on the sensory innervation from the hindbrain FSNs could be used to interrogate the evolution of forelimb sensorimotor systems.

## Data Availability Statement

The raw data supporting the conclusions of this article will be made available by the authors, without undue reservation.

## Ethics Statement

The animal study was reviewed and approved by the University of Chicago’s Institutional Animal Care and Use Committee.

## Author Contributions

KH, EM, and MH conceived of the experiments. KH conducted the experiments. AR and KH analyzed the data. AR, KH, and MH wrote the manuscript. All authors contributed to the article and approved the submitted version.

## Conflict of Interest

The authors declare that the research was conducted in the absence of any commercial or financial relationships that could be construed as a potential conflict of interest.

## Supplementary Material

Supplementary Figure 1 **(A)** A subset of HB FSNs exhibit a variety of innervation patterns on the axial surface. In some cases there is very little axial innervation, and in other cases there is extensive innervation in the immediate area of the fin. Cells are numbered according to putative clusters, but the grouping are small and unstable. **(B)** Cluster dendrogram and cluster plot (below) for the whole cell reconstructions in **(A)**. **(C)** Box plots of each of the 10 morphological parameters, together with two soma parameters, utilized in the cluster analysis. Boxes are color coded in accordance with cluster number. Black point overlays indicate the individual values for each neuron. Anterior is to the left and dorsal is up in **(A)**.

Supplementary Table 1 The minimum value, maximum value, mean, median, standard deviation, and *p*-value from Shapiro-Wilk Normality test of the 11 morphological parameters. *P*-values greater than 0.05 indicate normalcy, and the final column has a ^∗^ to highlight normally distributed parameters.

Supplementary Table 2 Full description of the morphological parameters.

Supplementary Table 3 Cell ID and cluster assignment number associated with soma position for plotting the *x*-axis in Figure 5.

Supplementary Movie 1 A 3D volume reconstruction of a single FSN exhibits the extensive innervation of the fin as well as the path of innervation along the surface of the skin. Anterior is to the right at the start of the movie, dorsal is up. The reconstruction rotates 180 degree along the anteroposterior axis.
