# Supplementary figures and images for: Hindbrain and Spinal Cord Contributions to the Cutaneous Sensory Innervation of the Larval Zebrafish Pectoral Fin
Source: Front Neuroanat. 2020 Oct 20;14:581821. doi: 10.3389/fnana.2020.581821 (PMC7607007; doi:10.3389/fnana.2020.581821)

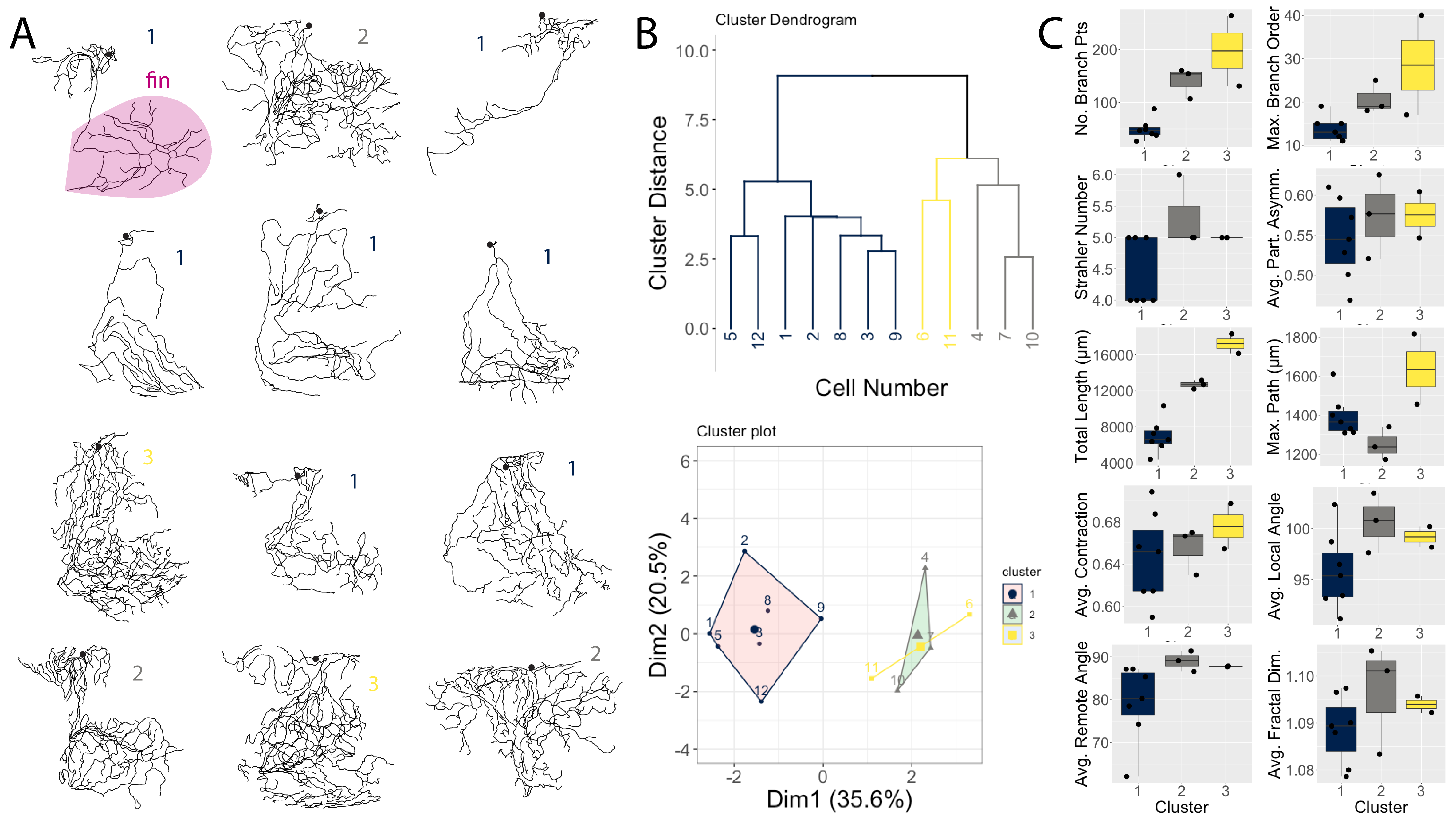

Supplement: Supplementary Figure 1 — (A) A subset of HB FSNs exhibit a variety of innervation patterns on the axial surface. In some cases there is very little axial innervation, and in other cases there is extensive innervation in the immediate area of the fin. Cells are numbered according to putative clusters, but the grouping are small and unstable. (B) Cluster dendrogram and cluster plot (below) for the whole cell reconstructions in (A). (C) Box plots of each of the 10 morphological parameters, together with two soma parameters, utilized in the cluster analysis. Boxes are color coded in accordance with cluster number. Black point overlays indicate the individual values for each neuron. Anterior is to the left and dorsal is up in (A). [file Image_1.tif]
