# Supplementary material for: Hindbrain and Spinal Cord Contributions to the Cutaneous Sensory Innervation of the Larval Zebrafish Pectoral Fin
Source: Front Neuroanat. 2020 Oct 20;14:581821. doi: 10.3389/fnana.2020.581821 (PMC7607007; doi:10.3389/fnana.2020.581821)
Supplement: Supplementary Table 1 — The minimum value, maximum value, mean, median, standard deviation, and p-value from Shapiro-Wilk Normality test of the 11 morphological parameters. P-values greater than 0.05 indicate normalcy, and the final column has a ∗ to highlight normally distributed parameters. [file Table_1.pdf]

Morphological Parameter	Min Value	Max Value	Mean	Median	SD	Shapiro-Wilk Normality p-value	Normal?
total length of innervation (µm)	971.92	7751.764	3409.5	3139.8	± 1857.869	0.019	
average contraction of the neuron (AU)	0.509	0.736	0.6252	0.642	± 0.066	0.121	*
number of branch points (AU)	1	83	23.5	15	± 20.92719	0.002	
maximum path distance (µm)	896.526	1748.026	1319.7	1315.8	± 202.6641	0.755	*
average branch fractal dimension (AU)	1.056	1.121	1.078	1.077	± 0.016	0.303	*
average partition asymmetry (AU)	0	0.875	0.483	0.509	± 0.198	0.371	*
average local angle (°)	57.0805	127.903	95.54	96.11	± 12.807	0.005	
average remote angle (°)	50.165	144.936	82.62	82.04	± 18.692	0.003	
maximum euclidean distance (µm)	349.222	699.373	491.2	467.3	± 89.653	0.227	*
Strahler number (AU)	2	5	3.75	4	± 1.070	0.002	
maximum branch order (AU)	2	15	9	0	± 3	0.612	*
