# Supplementary material for: Hindbrain and Spinal Cord Contributions to the Cutaneous Sensory Innervation of the Larval Zebrafish Pectoral Fin
Source: Front Neuroanat. 2020 Oct 20;14:581821. doi: 10.3389/fnana.2020.581821 (PMC7607007; doi:10.3389/fnana.2020.581821)
Supplement: Supplementary Table 2 — Full description of the morphological parameters. [file Table_2.pdf]

Morphological Parameter	Definition
total length of innervation (µm)	All of the segments added together.
average contraction of the neuron (AU)	Euclidean distance of each segment (total) / total length of the neuron. Ratio of euclidean distance to path length. Established space filling of neuron similar to 11), but through a different calculation. In this instance, one would be a total straight line (euclidean distance and path would be 1:1).
number of branch points	Purely the total number of branches of the afferents in the fin & the processes leading up to the fin (if they branch before entering the fin). This value gives an idea of how arborized the tree is.
maximum path distance (µm)	Following the longest afferent exactly from the soma to the terminal point.
average branch fractal dimension (AU)	Evaluates space filling, which can approximate how much info the primary afferent could detect. In our case, “how much the afferent meanders.” A straight line would be 1, and more meandering values are slightly higher values just above 1. This is averaged across all the branches of the afferents innervating the fin. In this value, we can again look at how much information can be encoded about the fin structure. If everything was a straight line through the fin, it would be unlikely that these cells would be encoding information about the fin surface. We do find a surprising number of the cells have a value closer to one, suggesting that there may be some specificity. We do not find any general trends though.
average partition asymmetry (AU)	A measure of the asymmetry of the tree. A value of 0 is completely symmetric, a value of 1 is asymmetric. In our case, the values were averaged across all the segments of the primary afferents of a single cell. Formula is $\|n1 - n2\| / (n1 + n2 - 2)$ where n1 = number of terminals on the right and n2 = number of terminals on the left. This parameter is measuring the complexity based on the number of terminal points, and it does not involve the “order” of the branches as the Strahler number does. It is also a measure of symmetry, but from a slightly different perspective.
average local angle (°)	This we calculated in the immediate area of the branch point, 10 nodes (aka pixels) away from the branch point on each segment. This value gives us an idea of the local coverage of the neuron. Again, this provides us some information about the amount of area that could be encoding sensory information.
average remote angle (°)	Same as above, but at the next branch down. This we decided rather arbitrarily because we wanted to be far enough away from local angle to be valuable, but not so far that it was meaningless in terms of angle.
Strahler number	Each terminal branch is labeled 1. Going back towards the soma on each branch, the next branch is n + 1 if BOTH daughters are labelled n. If one is n and the other is < n, it is also n. The number of the root segment (the initial one from the segment) is the Strahler number of the whole tree. The Strahler number gives us an idea of the geometry of the tree. So it gives us an idea of the complexity of the tree rather than just the pure branch order number. None of the parameters thus far tell us anything about the extent of coverage.
maximum branch order	Starting at the soma, how many branch points (n) are between the soma and the end point of the most branched afferent. Final value is n + 1. This value tells us the maximum extent of branching on the longest afferent. Functionally, this gives us an idea of how branched bigger neurons are. This information then allows us to make inferences about the amount of sensory information encoded by the neuron.
