# Supplementary material for: Hindbrain and Spinal Cord Contributions to the Cutaneous Sensory Innervation of the Larval Zebrafish Pectoral Fin
Source: Front Neuroanat. 2020 Oct 20;14:581821. doi: 10.3389/fnana.2020.581821 (PMC7607007; doi:10.3389/fnana.2020.581821)
Supplement: Supplementary Table 3 — Cell ID and cluster assignment number associated with soma position for plotting the x-axis in Figure 5. [file Table_3.pdf]

Cell ID	Cluster ID	Microns from Myomere 3/4 Boundary
14	2	-148.408
19	1	-98.992
6	2	-95.36
10	2	-93.16
8	1	-76.023
20	1	-73.261
5	1	-56.476
17	1	-56.035
16	1	-53.548
3	1	-45.347
12	1	-41.302
15	2	-31.801
7	1	-16.003
18	1	-13.666
13	1	-10.566
2	1	-8.051
4	1	-2.763
9	2	3.46
N/A	N/A	40.795
1	1	87.965
11	3	121.942
